# Differential Assessment of Internal Jugular Vein Stenosis in Patients Undergoing CT and MRI with Contrast

**DOI:** 10.3390/tomography10020021

**Published:** 2024-02-11

**Authors:** Mohamad Abdalkader, Matthew I. Miller, Piers Klein, Ferdinand K. Hui, Jeffrey J. Siracuse, Asim Z. Mian, Osamu Sakai, Thanh N. Nguyen, Bindu N. Setty

**Affiliations:** 1 Department of Radiology, Boston Medical, 840 Harrison Ave., Boston, MA 02118, USAasim.mian@bmc.org (A.Z.M.); osamu.sakai@bmc.org (O.S.); thanh.nguyen@bmc.org (T.N.N.); bindu.setty@bmc.org (B.N.S.); 2 Department of Medicine, Cambridge Health Alliance, Cambridge, MA 02139, USA; matmiller@challiance.org; 3 Neuroscience Institute, The Queen’s Medical Center, Honolulu, HI 96813, USA; ferdinandhui@gmail.com; 4 Department of Radiology, University of Hawaii, Honolulu, HI 96813, USA; 5 Department of Surgery, Boston Medical Center, Boston, MA 02118, USA; jeffrey.siracuse@bmc.org

**Keywords:** Magnetic Resonance Imaging, Computed Tomography Angiography, Jugular Veins

## Abstract

Objective: Internal Jugular Vein Stenosis (IJVS) is hypothesized to play a role in the pathogenesis of diverse neurological diseases. We sought to evaluate differences in IJVS assessment between CT and MRI in a retrospective patient cohort. Methods: We included consecutive patients who had both MRI of the brain and CT of the head and neck with contrast from 1 June 2021 to 30 June 2022 within the same admission. The degree of IJVS was categorized into five grades (0–IV). Results: A total of 35 patients with a total of 70 internal jugular (IJ) veins were included in our analysis. There was fair intermodality agreement in stenosis grades (κ = 0.220, 95% C.I. = [0.029, 0.410]), though categorical stenosis grades were significantly discordant between imaging modalities, with higher grades more frequent in MRI (χ^2^ = 27.378, *p* = 0.002). On CT-based imaging, Grade III or IV stenoses were noted in 17/70 (24.2%) IJs, whereas on MRI-based imaging, Grade III or IV stenoses were found in 40/70 (57.1%) IJs. Among veins with Grade I-IV IJVS, MRI stenosis estimates were significantly higher than CT stenosis estimates (77.0%, 95% C.I. [35.9–55.2%] vs. 45.6%, 95% C.I. [35.9–55.2%], *p* < 0.001). Conclusion: MRI with contrast overestimates the degree of IJVS compared to CT with contrast. Consideration of this discrepancy should be considered in diagnosis and treatment planning in patients with potential IJVS-related symptoms.

## 1. Introduction

Internal Jugular Vein Stenosis (IJVS) has been associated with diverse neurological conditions, including headache, brain fog, dizziness, tinnitus, transient global amnesia, and intracranial hemorrhage [1,2,3,4,5,6,7,8,9]. Accurate assessment of the internal jugular (IJ) vein via cross-sectional imaging such as CT and MRI is essential to delineate anatomical slenderness, incidental narrowing, and clinically significant stenoses in patients with potential IJVS-related symptoms. However, prior studies have demonstrated that IJVS may be present in up to two-thirds of unselected patients [10,11,12], thus complicating the process of diagnosing patients with true IJVS-related pathology and likely leading to low rates of recognition among neuroradiologists. Furthermore, the diagnosis of IJVS is hampered by ambiguous and often conflicting radiological assessments as differing imaging modalities may yield substantially divergent estimates of IJ calibers in the same patient [1]. MRI brain with contrast, including volumetric high-resolution T1 post-contrast sequence, is a commonly performed neuroradiological exam to evaluate a wide spectrum of neurological conditions, including IJVS-related symptoms. These high-resolution images offer valuable insights not only into brain parenchyma evaluation but also into assessing the intracranial venous system [13]. Typically, routine volumetric T1 post-contrast images encompass the IJ up to the C4–C5 cervical vertebra. However, the effectiveness and accuracy of this imaging sequence in evaluating the upper jugular veins have not been investigated. Moreover, in routine clinical practice at our institution, we have observed that contrast-enhanced MRI frequently exaggerates the extent of IJVS compared to CT scans. In this study, we aimed to compare brain MRI brain with contrast and contrast-enhanced CT (CT angiography and/or CT venography) findings of IJVS.

## 2. Methods

Institutional review board approval was obtained at the study site. The requirement to obtain written informed consent was waived. Anonymized data are available upon reasonable request to the corresponding author.

### 2.1. Patient Selection

Consecutive patients were retrospectively collected between 1 June 2021, and 30 June 2022, if they (1) underwent a contrast-enhanced CT scan of the head and the neck (CT angiography and/or CT venography), (2) underwent an a brain MRI brain with contrast study that included volumetric post-contrast imaging covering the upper neck (at least to the C4–C5 level) (3) both studies were performed within the same admission. Patients were excluded if the image quality was inadequate (motion or metal artifacts, incomplete contrast opacification of the venous system, incomplete imaging coverage of the upper neck), or the patient had either a thrombosis of the IJ or a prior history of neck surgery or radiation.

### 2.2. Scanning Protocols

All examinations were performed during routine clinical care and in accordance with local institutional protocols. All patients were scanned in a neutral supine position which was confirmed on the scout views of CT and MRI studies of all patients. Our local scanning protocols do not specifically direct patients to exhale or inhale during any particular time of image acquisition.

Contrast-enhanced CT scan was performed in single-phase acquisition using 64-detector row CT scanners (Lightspeed VCT; GE Healthcare, Chicago, IL, USA) with soft tissue and bone algorithm reconstructions. Axial images of 0.625 mm size thickness covering the aortic arch to the vertex were obtained in the arterial and/or venous phase after intravenous injection of lopamidol Injection (80–120 mL).

MRI examinations were performed with and without contrast on either 1.5 or 3 Tesla Phillips scanners or 3 Tesla GE scanners. There were 17 patients who underwent 3 Tesla MRI. This included 15/17 using Ingenia scanners (Philips HealthCare, Andover, MA, USA) and 2/17 using GE healthcare scanners (Chicago, IL, USA). 18 patients underwent 1.5 Tesla MRI using Achieva scanners (Philips Healthcare, Andover, MA, USA). MRI of the brain included Three-Dimensional Magnetization Prepared Rapid Acquisition Gradient Echo (3-D MPRAGE) [14,15] pre- and post-contrast sequences, acquired in the sagittal plane, and reformatted in the axial and coronal planes. Intravenous contrast administration was performed with Prohance (Bracco) based on the patient’s weight. The standard Gardolinium-based contrast agent used in Brain MRI was 0.1 mmol/kg of body weight. Parameters for each scanner are presented in Table A1 (See in Appendix A).

### 2.3. Imaging Analysis

CT and MR studies were independently evaluated by two board-certified neuroradiologists with 5 and 15 years of experience, respectively. In case of disagreement, consensus was reached for a final decision, such that there was complete interrater agreement of all measurements used for the final analysis. The degree of IJVS was categorized into five grades: Grade 0: No Stenosis, Grade I: ≤24%, Grade II: 25% to 49%, Grade III: 50% to 74%, or Grade IV: 75% to 100%. The presence of IJVS was defined as Grade I or greater. Maximal IJVS was measured using the ratio of the narrowest IJ diameter to the diameter of the normal proximal IJ at the level of the jugular bulb, similar to the North American Symptomatic Carotid Endarterectomy Trial (NASCET) criteria on both MRI and CT in patients with at least Grade I IJVS [16]. The maximal stenosis on both the right and left sides was selected on sagittal images on both CT and MRI, and the IJ surface areas were measured at the corresponding axial section using the freeform surface measurement tool on a GE PACS workstation.

As we aimed to compare IJ appearance using CT and MRI, our analysis was also conducted on a per-vein basis, involving a total of 70 veins. For further assessment of cerebral venous outflow, jugular vein dominance, styloid process enlargement/stylohyoid calcification/ossification, venous collaterals, condylar emissary vein size, and signs of intracranial hypertension (empty sella turcica, optic nerve tortuosity, transverse sinus stenosis) or confluent white matter hyperintensities were assessed. Causes of the stenosis on CT (compression by the digastric muscle, styloid process compression, stylohyoid ligament, lateral C1 vertebra) were also identified. IJ dominance was assessed by visual comparison of vessel calibers. Styloid process enlargement or elongation, and stylohyoid ligament calcification were recorded in a binary fashion. Condylar and mastoid emissary veins were assessed by measuring the largest intraosseous diameter on the axial source images.

### 2.4. Statistical Analysis

Statistical analysis was performed using Python 3.7, using the stats models (version 0.10.1) and SciPy (version 1.5.2) libraries. Continuous variables were reported as mean with 95% CI if conforming to a Gaussian distribution and median (interquartile range) otherwise. Categorical variables were depicted as counts and percentages.

We first characterized the categorical IJVS grades within our entire study cohort so as to mirror the diversity of cerebrovascular conditions encountered in clinical practice. This analysis included all patients, including those with Grade 0 stenosis (2) We then characterized continuous IJVS values among those veins with at least Grade I stenoses (i.e., those whom we considered to have clinically significant IJVS). For this phase of analysis, we only included patients with Grade I–IV stenoses so as to avoid the spurious effects of including many patients with 0% stenosis within our mean IJVS calculations.

Differences in paired continuous variables were assessed by Wilcoxon Rank Sum testing, and differences in paired categorical variables were analyzed via modified McNemar testing. Differences in continuous variables across categorical stenosis grades were assessed with the ANOVA test and the post hoc Tukey test for pairwise comparison. Cohen’s kappa score was used as a metric of interrater reliability for categorical stenosis grades between modalities. Pearson’s coefficient was used as a measure of correlation for continuous IJVS values. Differences in paired categorical variables were analyzed via modified McNemar testing and differences in paired continuous variables were assessed by Wilcoxon Rank Sum testing. Missing data were not imputed. Significance was set at α = 0.05 for all tests.

## 3. Results

A total of 35 patients with a total of 70 jugular veins were included in our analysis. The median age was 45.0 years (IQR: [37–62.5]), and 68.6% (24/35) were female. Indications for serial imaging with MRI and CT were varied and included stroke symptoms (20/35, 57.1%), suspicion of intracranial hemorrhage (3/35, 8.6%), trauma (3/35, 8.6%), and altered mental status (2/35, 5.7%). Clinical indications for the remaining seven patients (20.0%) included dizziness, suspected aneurysm, and suspected mass (Table 1). All imaging investigations were normal on formal reading and there was no comment on the jugular veins.

Across all IJVS categories (0-IV), there was fair agreement in stenosis grades between CT and MRI (κ = 0.220, 95% C.I. = [0.029, 0.410]), though the categorical distributions of IJVS grades indeed differed significantly (χ^2^ = 27.378, *p* = 0.002). Overall, on CT-based imaging, 37/70 (52.9%) of IJs were found to have at least some degree of IJVS (Grade I–IV). Grade III or grade IV stenoses were noted in 17/70 (24.2%) IJs. On MRI-based imaging, 47/70 (67.1%) were found to have some degree of IJVS. Grade III and Grade IV stenoses were found in 40/70 (57.2%) IJs (Table 2).

**Figure 1:**
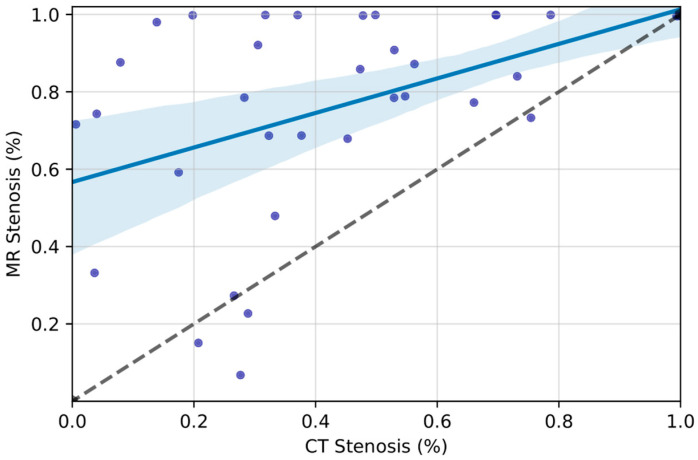
IJVS measurements, MRI vs. CT: Continuous IJVS measurements for veins with visual evidence of stenosis are compared. Each scatterplot marker corresponds to one distinct internal jugular vein. Best-fit line with 95% confidence interval is demonstrated. Only veins with at least Grade I-IV IJVS on both modalities are shown. Pearson coefficient (r = 0.477, 95% C.I. [0.189–0.703], *p* = 0.003) suggests moderate correlation between modalities in assessing IJVS, though there is marked divergence from an idealized symmetry between CT and MRI estimates (black dashed line).

**Figure 2:**
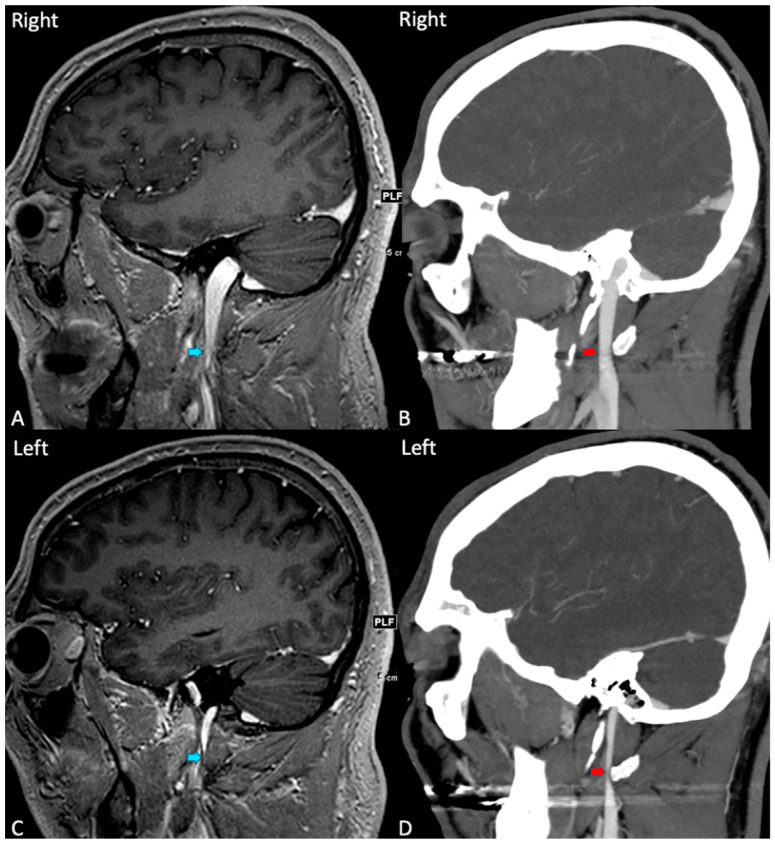
Apparent IJVS on MRI with vessel patency on CT venogram: Sagittal MRI brain with contrast ((**A**) on the right side and (**C**) on the left side) showing tapering and apparent stenosis of both internal jugular veins (blue arrows). Sagittal CT venogram with contrast ((**B**) on the right side and **D** on the left side) of the same patient showing normal size of the right internal jugular vein (red arrow, (**B**)) and moderate narrowing of the left jugular vein caused by the lateral mass of C1 (red arrow, (**D**)).

Among veins with Grade I–IV IJVS, MRI stenosis estimates were significantly higher than CT stenosis estimates (77.0%, 95% C.I. [35.9–55.2%] vs. 45.6%, 95% C.I. [35.9–55.2%], *p* < 0.001) (Figure 1, Table 3). The resulting categorical stenosis grades were significantly discordant by imaging modality, with higher grades more frequent by MRI (*p* = 0.002, Table 2, Figure 1). There was a moderate correlation between IJVS measurements on CT and MRI (r = 0.477, 95% C.I. [0.189–0.703], *p* = 0.003). Figure 2 demonstrates a patient with apparent severe stenosis of the right IJ on MR imaging but with a patent vessel on CTV, exemplifying the discordance observed in the greater cohort.

The right IJ was dominant in the majority of patients (21/35, 60.0%). The left jugular vein was dominant in nine patients (25.7%), and codominance was observed in five patients (14.3%). Bilateral enlargement of the styloid process was observed in ten patients (28.6%), and isolated right-sided stylohyoid ligament ossification was observed in one patient (2.9%). Most patients with stenoses did not have enlarged styloid processes or enlargement of C1 lateral processes on CT. The frequency of enlarged styloid processes/stylohyoid calcification did not differ significantly between patients with and without IJVS (*p* = 0.614). The intraosseous diameter of the condylar veins was not associated with the degree of IJVS on CT (r = 0.264, 95% C.I. [0.031–0.470] *p* = 0.145) or MRI (r = 0.043, 95% C.I. [−0.194–0.275] *p* = 0.796).

## 4. Discussion

In this single-center retrospective study, IJVS estimates were significantly overestimated by brain MRI with contrast compared to CT with contrast. Despite fair interrater agreement in categorical measurements between these modalities [17], when using MRI, an additional 33% of IJs were diagnosed with severe (Grade III or IV) IJVS, and the mean measured stenosis was 31.4% higher (77% vs. 45.6% on CT). This discrepancy carries important considerations for diagnostic and interventional workflows in patients undergoing brain MRI with contrast, especially those with neurologic symptoms thought secondary to IJVS, as reliance upon stenosis measured on MRI alone is likely to overestimate the contribution of this finding to a patient’s presentation. This may lead to unnecessary investigations and work-ups, patient concerns and anxiety, and, more importantly, unnecessary invasive interventions or surgeries. Our work also reveals an important directionality in this relationship between modalities: namely, if there is an absence of visual IJVS on CT, it is unlikely to be accompanied by IJVS on MRI. However, any IJVS that is present on CT is near-certain to be exaggerated on MRI.

Our results build upon recent work that demonstrated discordance of IJVS measurement by CT relative to catheter venography [1]. England et al. presented a case series of patients with complete occlusion of the IJ on CT venography but patency on catheter venography, suggesting that CT venography can also overestimate vessel stenosis. In our study, MRI produced far higher estimates of vessel stenosis than CT, but both methods identified high rates of incidental IJVS, in line with previous work [10]. Though studies describing IJVS evaluation by duplex ultrasound (DUS) are still lacking, together, these results may suggest that all forms of non-invasive imaging intrinsically overestimate the severity of IJVS. Given that cross-sectional imaging precedes invasive angiographic studies in the vast majority of clinical settings, radiologists, surgeons, and neurointerventionalists should carefully consider differences in stenosis estimates by imaging modality when contextualizing new findings of apparent IJVS.

An additional important finding in our study, consistent with previous research by Jayaraman et al. [10], is that IJVS is a common incidental finding in patients undergoing imaging investigations for other medical reasons. This finding holds significant implications when evaluating patients who undergo cross-sectional imaging, irrespective of whether they exhibit symptoms related to IJVS or not. It underscores the fact that IJVS is not necessarily indicative of a pathological condition alone and should be interpreted in conjunction with clinical symptoms and other imaging investigations. Indeed, we believe that our work should be taken as a foundational step prior to further explorations of the relationship of apparent IJVS on imaging studies and the spectrum of symptoms that may potentially result from this condition.

The differences in the measurement of IJVS between CT and MRI are likely the result of the methods underlying each modality. Though MRI of the brain is a high-resolution modality for the evaluation of neurologic symptoms, we posit that the physics of MRI acquisition may bias the appearance of the upper IJ at the time of evaluation. On 3D T1 sequences (e.g., MPRAGE, SPACE, VIBE) in particular, the superiorly located aspects of the neck veins may appear at the periphery of the field of view, leading to potential signal loss within venous structures [18]. Flow-related artifacts may also bias the luminal appearance of the IJ, as spatial misregistration may occur due to a mismatch between phase and frequency encoding within MR slices through which blood is actively moving (i.e., so-called voxel dephasing) [19,20]. Differing signal-intensity thresholds for maximum intensity projections (MIPs), along with partial volume averaging, may also lead to overestimation of vascular stenoses [21,22,23]. Even in the absence of motion degradation or susceptibility artifacts from adjacent skull base structures, these factors may lead to apocryphal evaluation of IJVS on MRI [24]. Conversely, prior arterial studies have suggested that CT is less susceptible to the overestimation of vascular stenoses given that it is an anatomically weighted modality with comparatively lower voxel size and slice thicknesses than MRI and thus significantly greater resolution [20]. The continued development of multidetector CT protocols is likely to further develop the trend toward greater accuracy in CT imaging, including for vascular imaging [25,26].

In addition to the technical imaging factors that contribute to inaccuracies in cross-sectional imaging, there are multiple anatomical factors that may affect the appearance of the IJ on such imaging. For instance, unlike the relatively stable arterial system, the venous system exhibits various variations, particularly in extracranial and cervical venous outflows. These variations include the anatomical slenderness of the IJ [11], changes in flow within the venous sinuses between standing and sitting positions [27], neck flexion versus extension or rotation positions, the patient’s volume status, and the patency of paravertebral and suboccipital collaterals. Additionally, the IJ can be easily displaced or compressed by adjacent anatomical structures such as the styloid process, stylohyoid ligament, posterior belly of the digastric muscle, internal or external carotid artery branches, and the lateral masses of C1 [3,10,12]. Additionally, inhalation and exhalation (e.g., Valsalva maneuver) are known to exhibit notable effects on the apparent caliber of the IJ [28]. However, given that our institutional protocols do not require inhalation or exhalation at specific points of the MR and CT imaging studies compared in this work, we feel that the effects of respiratory motion on IJ appearance are likely to be randomly distributed between CT and MRI in our cohort. As a result, we do not feel that one modality is biased more toward vein collapse versus the other.

Despite all of the factors that can contribute to significant variations in the extracranial venous system and its appearance on imaging, accurate depiction of IJVS is of crucial importance in various neurologic disorders where venous congestion may be a key mechanistic contributor to a patient’s clinical presentation. Reliable estimates of luminal patency, as well as greater awareness of imaging pitfalls in assessing it, may help to ensure timely identification of true pathologic states while also avoiding diagnostic cascades that prolong patient discomfort and contribute to the poor utilization of medical resources.

Based on this work, in cases where stenosis is discovered on CT scans in patients with potential IJVS-related symptoms, we suggest that further investigations such as angiogram/venogram with venous manometry would be necessary. On the other hand, if the stenosis is detected on MRI in patients with possible IJVS-related symptoms, a CT venogram would be an appropriate non-invasive next step in the management process. An idealized approach will likely utilize both modalities in a complementary fashion.

Our study demonstrates the presence of differential estimation of IJVS by CT and MRI but has important limitations. First, this was an observational retrospective single-center study with a relatively small sample size and strict exclusion criteria. All imaging studies we evaluated were conducted during routine clinical care, and we excluded all patients with possible IJVS-related symptoms or abnormal imaging findings on official radiology reports or with previous neck surgery or radiation so as not to confound our measurements. Second, the patients comprising our cohort did not undergo invasive imaging such as catheter-based angiography or venography, which would establish gold-standard measurements of intrinsic stenosis. In addition, in contrast to CT imaging that included dedicated imaging of the neck, the assessment of jugular veins on MRI was solely performed using brain MRI with contrast and not through a dedicated neck MRI with contrast or MR venogram of the neck. However, considering that the investigation of any neurological symptoms, whether associated with jugular stenosis or not, typically involves imaging of the brain using MRI with and without contrast, it becomes essential to have a comprehensive understanding of the IJ’s appearance and pitfalls on MRI brain imaging. This knowledge is crucial for preventing false diagnoses.

Lastly, as all of our patients had both a negative CT and MR, there was no clinical indication for catheter angiography or duplex ultrasound (DUS) within any of the cases included in our cohort. Although prior studies have demonstrated the high concordance of CT with both angiography and DUS for luminal imaging and stenosis quantification of the carotid arteries [20,21,29,30,31,32], future research directly comparing MRI, CT, angiography, and DUS is necessary in order to understand the full correlation between different imaging modalities in assessing IJVS and resultant symptomology. Additional work directly comparing patients with potential IJVS-related symptomology (e.g., those with pulsatile tinnitus) to asymptomatic patients should also be pursued so as to clarify the proper usage of imaging in diagnosing and ultimately treating these challenging conditions.

## 5. Conclusions

Brain MRI with contrast overestimates IJVS compared with CT, but incidental jugular stenosis is common in both modalities. This discrepancy carries significant implications for patients undergoing brain MRI with contrast, as it may lead to unnecessary invasive interventions or surgeries. Future prospective studies directly comparing MRI, CT, and venography are necessary to better comprehend the correlation between different imaging modalities in assessing IJVS and its clinical significance.

## Figures and Tables

**Table 1:** Cohort details: Clinical details are displayed for the study population, including demographics, clinical indications for imaging, and MR scanner type.

Characteristic.	N (%)
Female	24 (68.6)
Age (median, IQR)	45.0 (37–62.5)
Indication	
Stroke	20 (57.1)
ICH	3 (8.6)
Trauma	3 (8.6)
AMS	2 (5.7)
Other	7 (20.0)
MR Scanner	
1.5 T Philips	18 (51.4)
3 T Philips	15 (42.8)
3 T GE	2 (5.7)

**Table 2:** Categorical IJVS by laterality: IJVS grades for all IJs are compared between CT and MRI. Categorical distributions differ significantly between modalities according to modified McNemar testing (χ^2^ = 27.378, *p* = 0.002), and there is a tendency toward higher stenosis grading on MRI versus CT.

IJVS Grade	Right	Left	Total
CT			
Grade 0 N (%)	17 (48.6)	16 (45.7)	33 (47.1)
Grade I N (%)	2 (5.7)	4 (11.4)	6 (8.6)
Grade II N (%)	9 (25.7)	5 (14.3)	14 (20.0)
Grade III N (%)	5 (14.3)	5 (14.3)	10 (14.3)
Grade IV N (%)	2 (5.7)	5 (14.3)	7 (10.0)
Total	35	35	70
MRI			
Grade 0 N (%)	12 (34.3)	11 (31.4)	23 (32.9)
Grade I N (%)	3 (8.6)	1 (2.9)	4 (5.7)
Grade II N (%)	1 (2.9)	2 (5.7)	3 (4.3)
Grade III N (%)	2 (5.7)	7 (20.0)	9 (12.9)
Grade IV N (%)	17 (48.6)	14 (40.0)	31 (44.3)
Total	35	35	70

**Table 3:** Imaging characteristics of IJVS: Imaging characteristics are displayed for all IJs meeting at least Grade I stenosis criteria. Degree of stenosis, minimum surface area, and jugular bulb diameter are summarized.

Variable	CT	MR	*p*
IJs with Stenosis N (%)	37 (52.9)	47 (67.1)	0.28
IJVS Degree (%, 95% CI)	45.6 (35.9–55.2)	77.0 (67.8–86.1)	<0.001
Minimum Surface Area (mm^2^)	29.7 (23.1, 35.9)	11.5 (7.08, 15.8)	<0.001
Jugular Bulb Diameter (mm^2^, 95% CI)	55.8 (48.0, 62.2)	59.5 (52.6, 66.0)	0.39

## Data Availability

Data are available upon reasonable request from the corresponding author.

## Abbreviations

IJVS	Internal Jugular Vein Stenosis
IJ	Internal Jugular

## Appendix A

**Table A1:** MR Sequence Details.

	Scanner		
Property	1.5 T Philips	3 T Philips	3 T GE
*T2-STIR*			
TR (ms)	6000	8500	7800
TE (ms)	90	80	83
ST (mm)	5	5	5
SS (mm)	5	5	5
EN	1	1	1
*DWI*			
B	1000	1000	1000
TR (ms)	4500	4125	8500
TE (69)	69	86	77
ST (mm)	5	5	5
SS (mm)	5.5	5.5	5
EN	1	1	1
*FLAIR*			
TR (ms)	6000	8000	9800
TE (69)	100	135	141
ST (mm)	5	5	5
SS (mm)	5	5	5
EN	1	1	1
*SWI*			
TR (ms)	52	31	53
TE (69)	0	0	23
ST (mm)	3	3	3
SS (mm)	1.5	1.5	1.5
EN	1	1	1
*3D-MPRAGE*			
TR (ms)	8	8	10
TE (69)	4	4	4
ST (mm)	1.8	1	1
SS (mm)	0.9	1	0.5
EN	1	1	1
*T1 post*			
TR (ms)	650	360	425
TE (69)	10	4	15
ST (mm)	5	5	5
SS (mm)	5	5	5
EN	1	1	1
